# Becker’s nevus syndrome: a case report 

**DOI:** 10.1186/s13256-021-02996-y

**Published:** 2021-08-09

**Authors:** Ugo N. Chikani, Ijeoma N. Ohuche, Ada I. Bisi-Onyemaechi

**Affiliations:** Endocrine unit, Department of Paediatrics, University of Nigeria, Ituku Ozalla, Enugu, Nigeria

**Keywords:** Breast, Becker nevus, Syndrome

## Abstract

**Background:**

Becker’s nevus syndrome is a syndrome characterized by the presence of a Becker’s nevus with ipsilateral breast hypoplasia or hypoplastic defects of the muscle, skin, or skeleton. The nevus usually consists of a circumscribed, unilateral, irregularly shaped hyperpigmented macule, commonly occurring around the anterior upper trunk, with/without hypertrichosis and/or acneiform lesions. This rare syndrome has not been reported in our locality to the best of our knowledge.

**Case presentation:**

We report the case of a 15-year-old Igbo female patient who presented to our pediatric endocrinology clinic, University of Nigeria Teaching Hospital, Enugu, with complaints of asymmetry of the breasts and hyperpigmented macules on the side. Based on her symptoms, diagnosis of Becker’s nevus syndrome was made. The diagnosis of Becker’s nevus syndrome is mostly clinical, based on the presence of a Becker’s nevus with ipsilateral breast hypoplasia or hypoplastic defects of the muscle, skin, or skeleton. In our patient, there was a Becker’s nevus with ipsilateral breast hypoplasia. This syndrome, belonging to the class of epidermal nevus syndromes, is very rare, and is usually benign. She was placed on spironolactone tablets 50 mg daily, which have been associated with an improvement in the size of the hypoplastic breast, and her fears were allayed.

**Conclusion:**

This syndrome has not been reported in our locality to the best of our knowledge and, therefore, has a propensity for misdiagnosis by clinicians because of its rarity. We therefore report this to create awareness among clinicians regarding this condition that is associated with much psychosocial trauma among patients, and that can be easily managed with oral spironolactone.

## Introduction

Becker’s nevus syndrome (BNS) is a rare syndrome, belonging to the class of epidermal nevus syndromes, that is characterized by the presence of a Becker’s nevus with ipsilateral breast hypoplasia or hypoplastic defects of the muscle, skin, or skeleton [1]. The nevus usually consists of a circumscribed, unilateral, irregularly shaped hyperpigmented macule, commonly occurring around the anterior upper trunk, with/without hypertrichosis and/or acneiform lesions [1]. Histologically, the epidermis demonstrates acanthosis and rete ridge elongation in association with increased pigment in the basal cell layer and melanophages in the upper dermis. Smooth muscle bundles in the dermis may be increased in some cases, reminiscent of changes observed in congenital smooth muscle hamartomas [1]. Becker’s nevus was first described by Becker in 1949 [2], and in 1995 [3], Becker’s nevus syndrome was described by Happle as the occurrence of Becker’s nevus in association with unilateral breast hypoplasia and muscle, skin, and/or skeletal abnormalities. This syndrome is also known as hairy epidermal nevus syndrome [2].

An anti-androgenic agent, spironolactone, has been used in the medical treatment of BNS subsequent to histopathological finding of high levels of androgen receptors in the Becker’s nevus, which was first reported in 1984 [4]. Laser treatment is used for the removal of associated hyperpigmentation [1].

This rare syndrome has not been reported in our locality to the best of our knowledge, A systematic review by Schneider *et al.* in 2016 observed that almost half of the case reports were written by European authors (46%), Some cases were also reported in North and South America, as well as in Asia, but none from Africa, let alone Nigeria [5], hence the compelling need to report the case of a patient who recently presented to our clinic. We call the attention of clinicians to this rare cause of worry and anxiety among adolescents and their caregivers.

We also highlighted the reliance on clinical acumen for diagnosis seen in our country and, indeed, many other developing countries, where out-of-pocket expenditure for health care services makes laboratory diagnosis a luxury in many cases.

## Case presentation

A 15-year-old Igbo female from Enugu, Nigeria, presented in our clinic with her grandmother, with a history of asymmetry of the breasts noticed 4 years ago. The right breast was noticed to be gradually increasing in size, to its present adult size, with no corresponding change in the size of the left breast. There was no preceding history of trauma, and no nipple discharge. The obvious asymmetry had been a constant source of anxiety and embarrassment for the patient, and also for her caregiver. They had sought treatment at various health facilities but presented to our clinic eventually. The past medical was not remarkable, and she was not on any previous medications. She achieved menarche at 13 years of age. Both parents are dead; she lives with the grandmother, and they are of low socioeconomic status. At presentation, they had intense fears of the need for a plastic surgical correction of the left breast.

**Fig. 1:**
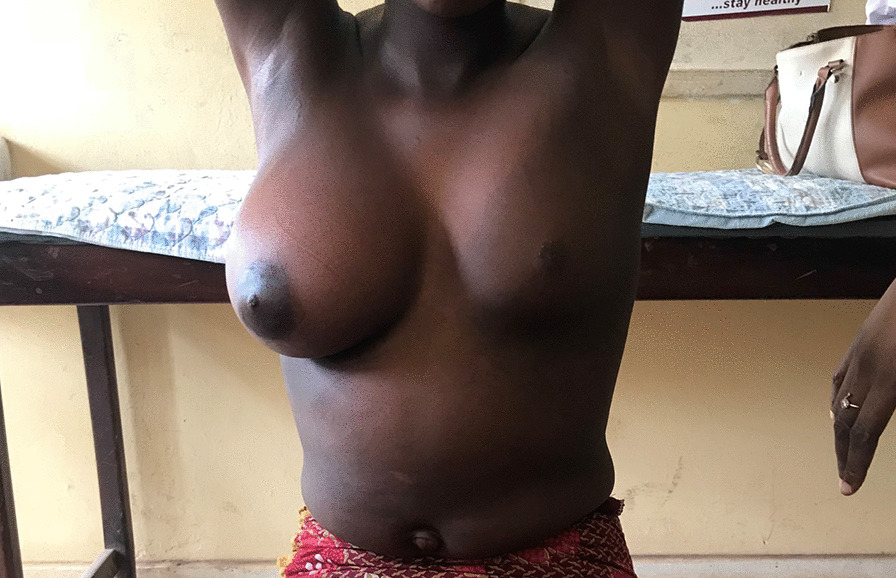
Before treatment

**Fig. 2:**
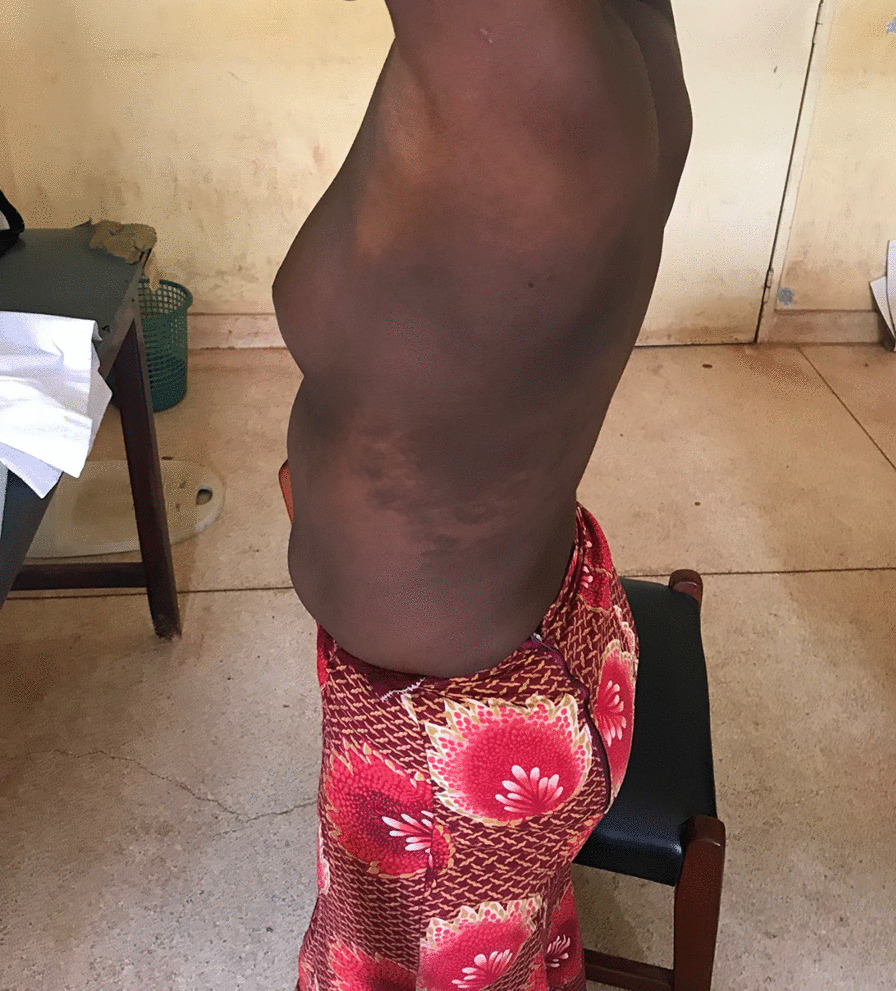
Lateral view

On examination, she was apprehensive and visibly self-conscious; her vital signs were stable with temperature of 36.8 °C, pulse rate of 80 beats per minute and blood pressure of 90/60 mmHg. She had marked breast asymmetry, with hypoplasia of the left breast of SMR 11 (Figs. 1 and 2), while the right breast had a staging of SMR V. In addition, we noted a large, hyperpigmented patch extending from the left axilla and covering the left anterior chest wall, and spreading posteriorly. This had been present since birth, and had not been given much attention since it was not associated with pain or other asymmetric skeletal abnormalities. Neurological examination did not reveal any abnormality. Abdominal ultrasound and skeletal radiographic imaging were unremarkable.

**Fig. 3:**
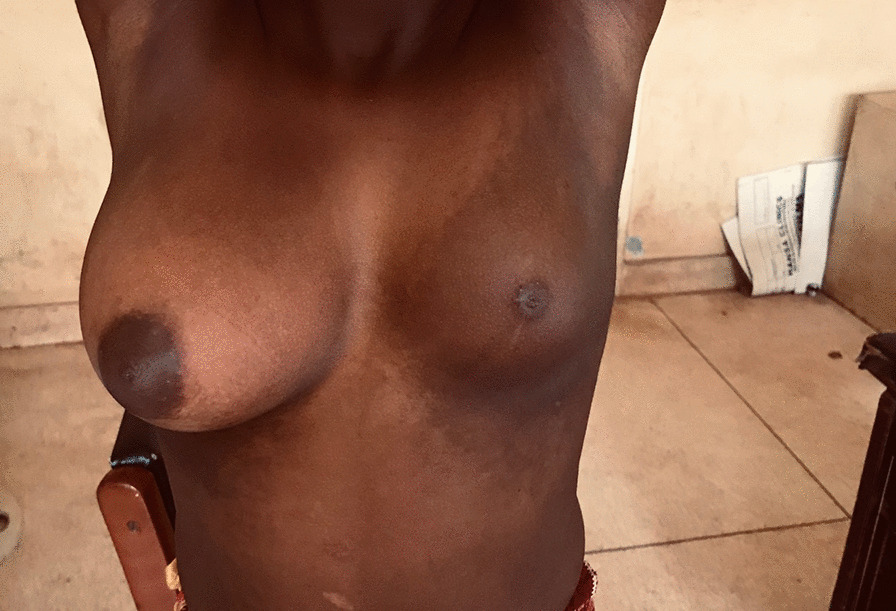
Still on treatment

Based on her symptoms, diagnosis of Becker’s nevus syndrome was made. The diagnosis of Becker’s nevus syndrome is mostly clinical, based on the presence of a Becker’s nevus with ipsilateral breast hypoplasia or hypoplastic defects of the muscle, skin, or skeleton. In our patient, there was a Becker’s nevus with ipsilateral breast hypoplasia. This syndrome, belonging to the class of epidermal nevus syndromes, is very rare, and is usually benign. She was placed on spironolactone tablets (50 mg daily). After 6 months of therapy, there was an improvement in the size of the hypoplastic breast; increased from SMR 11 to SMR 111 (Fig. 3), and her fears were allayed.

## Discussion

This case was peculiar because it occurred in a female, whereas the majority of cases occurred in males [4, 5], and the nevus was noticed since birth and on the ipsilateral side with the hypoplastic breast. In addition, the syndrome has not been reported in our locality to the best of our knowledge and has a propensity for misdiagnosis by clinicians because of its rarity. We therefore report this to create awareness among clinicians regarding this condition that is associated with so much psychosocial trauma among patients, and that can be easily managed with oral spironolactone.

Becker’s nevus syndrome is a rare syndrome, and the diagnosis is mostly clinical, characterized by a well-outlined area of hyperpigmentation, in association with hypoplasia of the ipsilateral breast, areola and/or nipple, musculature, adipose tissue, and limb [4]. The diagnosis is also supported by histology finding of high levels of androgen receptors in the hypoplastic muscles comparable to the levels expressed at the genitals. Biopsy of the skin lesion revealed an increase in the number of smooth muscle bundles, hair follicles, and enlarged papillary crests with pigmentation in basal epidermis [6]. 

A systematic review of 84 cases of BNS reported ipsilateral breast hypoplasia as the most commonly described malformation associated with BNS and was observed in 56% (*n* = 47) of cases [5]. Diagnosis of our case and institution of treatment were solely based on clinical criteria, which included ipsilateral hypoplastic breast and hyperpigmented macules. Laboratory and histological studies of the tissue were not done because of the aforementioned financial challenges However, the response to treatment was similar to other reports [7–10] that started treatment based on both clinical diagnostic criteria and histology findings.

Our case responded remarkably to 50 mg of spironolactone within 6 months of therapy, which is in agreement with Hoon *et al.* [11], who also reported remarkable improvement in a case after 50 mg of spironolactone was administered daily. One month later, breast enlargement was seen only in the hypoplastic breast with the Becker’s nevus.

This syndrome has been shown to be androgen dependent, accounting for a higher prevalence in men and in adolescence, and some studies have demonstrated an increase in the number of androgen receptors associated with the Becker’s nevus [11]. Even though the pathogenesis of the associated breast hypoplasia remains unclear, it is hypothesized that increased androgen receptors in the breast may antagonize the stimulatory effect of estrogen on breast development, resulting in hypoplasia [10]. This symptom has been shown to respond to oral administration of spironolactone, an anti-androgen, further strengthening the hypothesis of increased androgen receptors in the hypoplastic breast [12]. 

This uncommon syndrome (less than a hundred cases reported in the last decade) has been reported more in females by some authors, with a female-to-male ratio of 1.5:1.0 [4]. The preponderance of cases in females may be due to the most commonly associated anomaly, breast hypoplasia, being more apparent in females [5], and is a cause of anxiety, as seen in the index report. However, some other authors have documented a higher male preponderance, with a male-to-female ratio of 5:1 [8]. Kim *et al.* [12] documented a slight male dominance among children less than 18 years of age, with equal sex ratio among children less than 14 years of age. Overall, it seems that, being an androgen-dependent disorder, this syndrome is more common in males, but more easily diagnosed in females [10]. The nevus constitutes the hallmark of the syndrome and is considered an androgen-dependent epidermal nevus, usually seen on the thorax or scapular region [11]. However, it may occur in any other part of the body, and may regress with time [10]. 

This nevus has similar preference sites in both children and adults, but is less commonly associated with hypertrichosis in pediatric patients than in adults [7]. Some studies have described hypertrichosis as seen more commonly in congenital smooth muscle harmatoma, a similar condition, than in Becker’s nevus [8].

Though the vast majority of documented cases appear sporadically, a familial grouping may be observed in very rare cases [13, 14], as a result of the predominant inheritance phenomenon. However, the genetic basis of this disorder is yet to be fully elucidated.

## Conclusion

Becker’s nevus is rare clinical syndrome, whose diagnosis is mainly clinical, associated with a lot of anxiety. The syndrome has been shown to respond to spironolactone.

## Data Availability

Not applicable.

## Abbreviations

BNS: Becker’s nevus syndrome
SMR: Sexual maturity rating
Fig: Figure
